# Prevention and treatment of long-term social disability amongst young people with emerging severe mental illness with social recovery therapy (The PRODIGY Trial): study protocol for a randomised controlled trial

**DOI:** 10.1186/s13063-017-2062-9

**Published:** 2017-07-11

**Authors:** David Fowler, Paul French, Robin Banerjee, Garry Barton, Clio Berry, Rory Byrne, Timothy Clarke, Rick Fraser, Brioney Gee, Kathryn Greenwood, Caitlin Notley, Sophie Parker, Lee Shepstone, Jon Wilson, Alison R. Yung, Joanne Hodgekins

**Affiliations:** 1 School of Psychology, Pevensey Building, University of Sussex, Falmer, Brighton, UK; 2 Psychosis Research Unit, Greater Manchester Mental Health NHS Foundation Trust, Manchester, UK; 3 School of Psychological Sciences, The University of Liverpool, Liverpool, UK; 4 Norwich Medical School, University of East Anglia, Norwich Research Park, Norwich, Norfolk UK; 5 Research & Development, Sussex Education Centre, Millview Hospital, Sussex Partnership NHS Foundation Trust, Nevill Avenue, Hove, BN3 7HZ UK; 6 School of Psychological Sciences, The University of Manchester, Oxford Road, Manchester, UK; 7 Research & Development, Norfolk and Suffolk NHS Foundation Trust, 80 St Stephens, Norwich, UK; 8 Brighton and Sussex Medical School, University of Sussex, Falmer, Brighton, UK; 9 Institute of Brain, Behaviour and Mental Health, The University of Manchester, Oxford Road, Manchester, UK

**Keywords:** Youth, Mental health, Social recovery, At risk mental states, Randomised controlled trial, Time use

## Abstract

**Background:**

Young people who have social disability associated with severe and complex mental health problems are an important group in need of early intervention. Their problems often date back to childhood and become chronic at an early age. Without intervention, the long-term prognosis is often poor and the economic costs very large. There is a major gap in the provision of evidence-based interventions for this group, and therefore new approaches to detection and intervention are needed. This trial provides a definitive evaluation of a new approach to early intervention with young people with social disability and severe and complex mental health problems using social recovery therapy (SRT) over a period of 9 months to improve mental health and social recovery outcomes.

**Methods:**

This is a pragmatic, multi-centre, single blind, superiority randomised controlled trial. It is conducted in three sites in the UK: Sussex, Manchester and East Anglia. Participants are aged 16 to 25 and have both persistent and severe social disability (defined as engaged in less than 30 hours per week of structured activity) and severe and complex mental health problems. The target sample size is 270 participants, providing 135 participants in each trial arm. Participants are randomised 1:1 using a web-based randomisation system and allocated to either SRT plus optimised treatment as usual (enhanced standard care) or enhanced standard care alone. The primary outcome is time use, namely hours spent in structured activity per week at 15 months post-randomisation. Secondary outcomes assess typical mental health problems of the group, including subthreshold psychotic symptoms, negative symptoms, depression and anxiety. Time use, secondary outcomes and health economic measures are assessed at 9, 15 and 24 months post-randomisation.

**Discussion:**

This definitive trial will be the first to evaluate a novel psychological treatment for social disability and mental health problems in young people presenting with social disability and severe and complex non-psychotic mental health problems. The results will have important implications for policy and practice in the detection and early intervention for this group in mental health services.

**Trial registration:**

Trial Registry: International Standard Randomised Controlled Trial Number (ISRCTN) Registry.

Trial Registration Number: ISRCTN47998710 (registered 29/11/2012).

**Electronic supplementary material:**

The online version of this article (doi:10.1186/s13063-017-2062-9) contains supplementary material, which is available to authorized users.

## Background

It is widely recognised that most socially disabling chronic and severe mental health problems begin in adolescence, with 75% of all severe and chronic mental illnesses emerging between the age of 15 and 25 years [1, 2]. Retrospective studies have consistently shown that severe mental illness is often preceded by social decline, which often becomes stable, and that such pre-morbid social disability is predictive of the long-term course of the disorder [3–9]. Between 3% and 5% of adolescents present with complex mental health problems associated with social disability [2]. The young people at highest risk of long-term social disability present with emerging signs of social decline, in association with low level psychotic symptoms, emotional and behavioural disorder, and often accompanied by substance misuse problems and risk of harm to self and others [1, 2]. Social disability in young people with severe mental health problems can be operationally defined behaviourally as low activity, or time spent, in social and economic domains [10]. The target population at high risk of long-term social disability and chronic mental health problems are therefore young people with severe and complex mental health problems who are not in education employment and training (NEET) and who are inactive and socially withdrawn. The severe and complex mental health problems typically include severe depression and anxiety symptoms often, but not always, accompanied by sub-threshold psychotic symptoms (paranoia and anomalous experiences) and frequently other symptomatic comorbidities (obsessive compulsive disorder, body dysmorphic symptoms and so on). This includes cases that meet formal diagnostic criteria for at risk or ultra-high risk for psychosis who are withdrawn and socially and economically inactive, but it does not exclude cases who have other types of severe and complex mental health problems associated with social disability. From the perspective of clinical staging [11], these may be regarded as severe cases at stage 1b having attenuated syndromes with mixed and ambiguous symptomatology and severe functional impacts.

The economic costs of not addressing mental health problems associated with social disability at an early stage are high [12]. Persistent mental health problems associated with social disability in young people often do not resolve naturally and may persist across the life course, resulting in severe distress and long-term social disability, as well as high costs in terms of use of health, social and other services [1, 2]. Health economic modelling of lifelong costs in this area are still emerging; however, one recent estimate suggests that mental health problems in childhood and adolescence can result in a 28% reduction in economic activity at age 50, with consequences across domains of marital satisfaction, self-esteem and quality of life leading to a £388,000 lifetime loss per person [13, 14]. Young people who have a combination of severe and persistent mental health needs and who are socially disabled present with problems that have the highest lifelong burden [14].

Despite poor outcomes and the high cost of disorders leading to social decline, young people with complex needs frequently do not access treatment. Recent estimates suggest that fewer than 25% of young people and their families who have needs gain access to specialist mental health services [15, 16]. Recent reports have highlighted that there is a major gap in identifying and managing severe and complex mental health problems in young people, particularly in those at risk of social disability [14–19]. Several UK NICE guidelines have highlighted this issue, including those for social anxiety [20] and depression [21], and the NICE guidelines on psychosis and schizophrenia in children and young people [17].

Young people with severe and complex mental health problems and who are socially disabled often fall between the net and do not access treatment. In the UK, such individuals tend not to be suitable for, or respond to, short-term evidence-based therapies for discrete mental health problems, such as cognitive behavioural therapy (CBT) for anxiety, depression and conduct disorder that are available via the Improving Access to Psychological Therapies initiative. However, they do not meet diagnostic criteria for specialist first episode psychosis services, for which there is now considerable evidence of benefits on social functioning [22–24]. If help seeking, their pathways to care are often diverse and, while some may receive care from Child and Adolescent Mental Health Services and Adult services, others are regarded as not meeting the diagnostic thresholds for entry or may disengage from services that do not offer assertive outreach. Many cases only have support from primary care despite having severe and complex mental health needs and social disability. The most systematic service provision is often outside mental health services in statutory and voluntary sector provision for young people who are NEET. In these services, the focus is primarily on obtaining employment and thus the mental health problems that present barriers to activity, work, education and training may not be recognised [25]. However, the degree to which NEET status is associated with mental health problems is being increasingly recognised [26–31]. Detection of such cases therefore needs to focus on screening of mental health problems of young people who have links with NEET services and who are under primary mental health care alongside seeking referrals from those referred into Child and Adolescent Mental Health Services and Adult services. Cases may also be detected in services for those in the at risk mental state (ARMS) for psychosis group, where these are present.

Current evidence for effective interventions to address social disability amongst young people in the early course of severe mental illness is limited [3]. A series of studies have been undertaken that have aimed to identify cases at ultra-high risk (UHR) of poor long-term outcome associated with severe mental illness, focusing predominantly on risk of psychosis [32–37]. These UHR studies have shown that it is possible to establish services to identify and treat cohorts of young people who can be identified as having ARMS using defined operational criteria and structured assessment tools [38]. Furthermore, these studies have consistently identified that those who are at the highest risk of poor outcomes are young people who present with social decline as well as sub-threshold psychotic symptoms [39, 40]. However, the focus of treatment trials in the UHR group has been on prevention of first episode psychosis, not social disability. A recent meta-analysis of intervention trials in this group found a reduction in risk of psychosis with specific treatments but no improvement in functioning compared to control conditions [41].

Systematic reviews of CBT for psychosis have consistently shown moderate effect size improvements in social disability where this has been assessed as a secondary outcome [42, 43]. However, these studies have predominantly been carried out amongst participants with chronic disorders rather than emerging problems. The feasibility of using CBT with young people who are at UHR of psychosis has been demonstrated by the Early Detection and Intervention Evaluation for People at Risk of Psychosis (EDIE 2) multicentre study [44], which has shown reductions in severity of psychotic symptoms. However, the primary focus of the therapy in EDIE 2 and other studies of cognitive therapy in UHR [17] was symptom reduction [45] and this approach neither targeted nor had a significant benefit on social disability. The present trial moves on from EDIE 2 by (1) focussing on a group of young people who are UHR and have problems defined by social disability (low structured activity levels) and (2) using a multi-systemic intervention that specifically aims to address social disability.

A previous multi-systemic social recovery therapy (SRT) intervention trial (Improving Social Recovery in Early Psychosis; ISREP) was carried out with a group of young people who had established chronic and severe social disability up to 8 years after a first episode of psychosis. This demonstrated gains in structured activity and hope as well as reductions in symptoms [46]. Health economic benefits were also demonstrated [47]. However, the trial was small and there was a high level of uncertainty associated with these estimates. A more recent study has confirmed that SRT can improve the activity of young people with first episode psychosis who had persistent social recovery problems [48], the effect was clearest at post treatment showing earlier social recovery and there were also promising indications of persistence of the effect albeit limited by missing data at follow-up. The SRT intervention tested in the present trial (PRODIGY) has been refined from experience in these previous studies to be applied to a novel population – socially disabled young people with severe and complex mental health problems [49]. Qualitative studies have confirmed the acceptability and satisfaction of participants with both PRODIGY trial procedures and SRT [50, 51]. Our approach is consistent with the goals of the recovery movement in that the focus is not just on symptoms but on wider outcomes, including social activities, work and education [52, 53]. PRODIGY will be the first trial to specifically test the effectiveness of an intervention that aims to improve both social recovery and mental health outcomes amongst a high-risk population of young people presenting with social disability and severe and complex mental health problems.

The present study aims to undertake a definitive randomised controlled trial (RCT) to determine the clinical and cost-effectiveness of SRT compared to enhanced standard care (ESC) in young people who present with social disability and severe and complex non-psychotic mental health problems, and who are at risk of long-term social disability and mental illness.

### Primary hypothesis

In young people who are socially disabled and have severe and complex non-psychotic mental health problems, SRT will be superior to ESC in improving social recovery (as measured by hours in structured activity assessed on the time use survey), at 15 months post-randomisation.

### Secondary hypotheses

First, that SRT will be superior to ESC in terms of cost-effectiveness and, second, that SRT will be superior to ESC in effects on mental health symptoms.

## Methods

### Trial design

PRODIGY is a pragmatic, multi-centre, single blind, parallel, superiority RCT comparing the clinical and cost-effectiveness of SRT delivered over a 9-month period plus optimised treatment as usual (ESC) against ESC alone. Participants are young people (aged 16 to 25 years) with severe and complex mental health problems and showing early signs of persistent social disability. The primary outcome is time use (weekly hours in structured activity) at the primary time point of 15 months. Time use and secondary outcomes will be evaluated at 9 and 15 months post-randomisation with 15 months as the primary endpoint; there will be a further assessment at 24 months post-randomisation to assess the persistence of the effect.

### Intervention

#### SRT

The intervention is SRT plus ESC delivered by trial therapists who are clinical psychologists or qualified CBT therapists trained in the intervention by the first and second authors (DF, PF). SRT is described in a therapy manual [49]. SRT is delivered individually in face-to-face sessions (Median = 15 sessions) across 9 months and may involve interim telephone and email contact. Sessions take place in participants’ homes, National Health Services (NHS) premises, community and public locations. All sessions, except where conducted in public locations, are audio-recorded with participant consent.

SRT is based on a CBT model which suggests that social disability evolves as a result of lifestyle patterns of low activity, which are adopted as functional behavioural patterns of avoidance and maintained by lack of hope, a reduced sense of agency and low motivation. The intervention involves promoting a sense of agency, hope and motivation, and encouraging activity while managing sub-threshold psychotic symptoms where present, non-psychotic mental health problems and neurocognitive functioning difficulties. The approach combines multi-systemic working with the use of specific CBT techniques. Multi-systemic working involves working with participants’ relatives and employment or education providers. Trial therapists adopt assertive outreach youth work principles and also draw from successful social and vocational interventions, such as supported education and employment interventions.

The intervention involves three stages that are flexibly tailored to each participant’s goals and problems:

- Stage 1 involves assessment and developing a formulation of the person’s difficulties and barriers to social recovery. This often involves validation and acceptance of real barriers, threats and difficulties, while focusing on promoting hope for social recovery.
- Stage 2 involves identifying and working towards medium- to long-term goals guided by a systemic formulation of barriers to recovery. A particularly important aspect of this is identifying specific pathways to meaningful new activities and values. Where relevant, this includes referral to appropriate vocational agencies, or alternatively direct liaison with employers or education providers. Additional specific techniques used in this stage include cognitive work promoting a sense of agency, consolidating a positive identity and addressing feelings of stigma and negative beliefs about self and others.
- Stage 3 involves the active promotion of social activity, work, education and leisure linked to meaningful goals, while managing symptoms. This involves specific cognitive behavioural techniques involving managing symptoms while promoting activity using behavioural experiments.

#### ESC

The control comparator is ESC alone. There is no restriction on access to existing NHS standard treatment for young people with non-psychotic severe and complex problems and social disability. ESC can include provision of short-term individual and family psychological therapies, medication management, support and monitoring within primary or secondary mental health services. Participants may also receive a range of education, social, training, vocational and youth work interventions from a variety of statutory and non-statutory service providers (including social services, voluntary agencies, employment and education providers). ESC also involves the provision of a Best Practice Manual for standard treatment from the trial team to the referrer and usual care provider (if not the referrer), which summarises good practice, including referral to mental health services and medication management where appropriate. The Best Practice Manual has been produced by monitoring and mapping service contacts received across a range of services in the population of interest.

Participants and referrers and/or the usual clinical team (with participant consent) also receive assessment feedback from the trial team pertaining to clinical and social circumstances. Assessments identify any risks to self or others and these are communicated to the referring clinicians to facilitate appropriate management. As identified in our qualitative studies [50, 51], participation in the trial in both trial arms is experienced by many participants as beneficial and an enhanced intervention. The Best Practice Manual and the approach of the trial team has been supported by service user groups and steering groups overseeing youth mental health provision in each of the regions, and its delivery has been well received by participating services, with referrers keen to involve participants in both treatment and control arms.

### Outcomes

#### Primary outcome

The primary outcome is time use (hours per week engaged in structured activity) measured at the 15 months post-randomisation. This assessment is derived from the Office of National Statistics Time Use Survey interview [54], adapted for use with a clinical population [10]. Number of hours per week engaged in structured activity includes time spent in both constructive economic activity, e.g. paid and voluntary work, education, childcare, housework and chores, and in structured activity, including leisure and sports.

#### Secondary outcomes

**Fig. 1:**
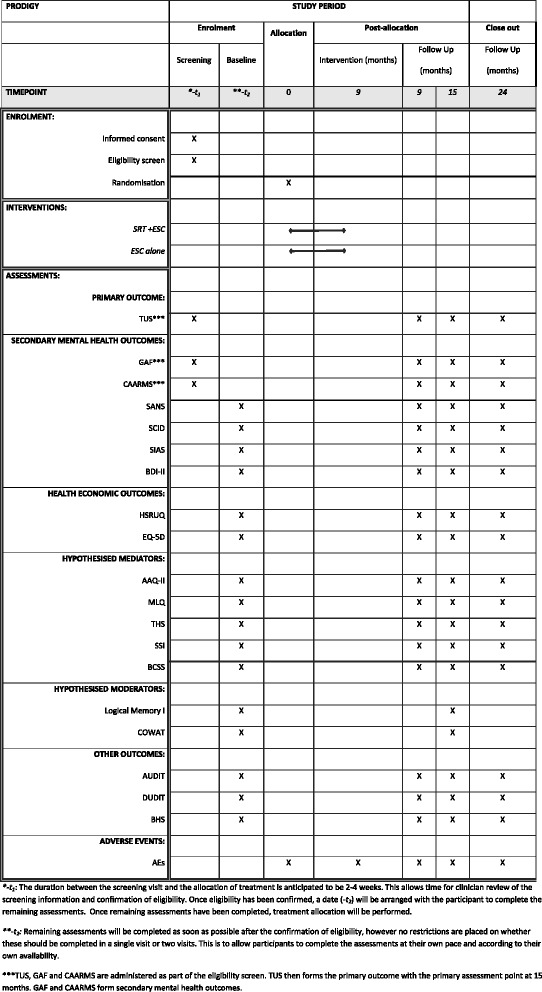
Standard Protocol Items: Recommendations for Interventional Trials (SPIRIT): Schedule of enrolment, interventions and assessments

Secondary outcomes focus on typical mental health problems of this group and include levels of attenuated psychotic symptoms and associated psychopathology using the Comprehensive Assessment of At Risk Mental States (CAARMS) interview [38], change in diagnostic status from Structured Clinical Interview for Diagnostic and Statistical Manual of Mental Disorders, Version Four [55], The Social Interaction Anxiety Scale [56] and Beck Depression Inventory-II [57], and the Scale for the Assessment of Negative Symptoms [58]. The assessments and the timing are outlined in the SPIRIT figure (Fig. 1). All outcomes will be assessed at baseline and again at 9, 15 and 24 months post-randomisation. The neurocognitive assessments are only assessed at baseline and 15 months post-randomisation. See the Additional file 1 for the SPIRIT checklist.

Mediators include the Acceptance and Avoidance II scale [59], Meaning in Life Questionnaire [60], Trait Hope Scale [61], Schizotypal Symptoms Inventory [62] and Brief Core Schema Scales [63].

Neurocognitive function will be assessed as a moderator and includes Logical Memory I subtest of the Wechsler Memory Scale, Third Edition [64] and Controlled Oral Word Association Test [65].

Other outcomes include Alcohol Use Disorders Identification Test [66], Drug Use Disorders Identification Test [67] and the Beck Hopelessness Scale [68].

#### Health economic outcomes

Costs will be estimated from the perspective of the NHS and personal social services (PSS) using the Health Service Resource Use Questionnaire [69] and quality of life will be assessed via the EuroQol-5D-3 L [70].

#### Adverse events

All serious adverse events and adverse events will be categorised and recorded from the point of randomisation until completion of the final follow-up assessment. All events will be reported to sponsor, local trial site, clinical trials unit (CTU), and trial oversight committees (Data Monitoring and Ethics Committee and Trial Steering Committee). Serious adverse events will be onward reported to the NHS ethics committee as appropriate.

### Participants

#### Inclusion criteria

- Young people aged 16 to 25 years with severe and complex mental health problems and showing early signs of persistent social disability.
- Presence of impairment in social and occupational function indicated by patterns of structured and constructive economic activity of less than 30 hours per week and a history of social impairment problems lasting for a period of longer than 6 months.
- Presence of severe and complex mental health problems defined operationally as:
- having attenuated psychotic symptoms which meet criteria for an ARMS, or
- having severe and complex mental health problems which score 50 or below on the Global Assessment of Function Scale (which indicates the presence of severe symptoms of at least two of depression, anxiety, substance misuse, behavioural or thinking problems, or subthreshold psychosis to the degree to impair function) with at least moderate symptoms persisting for longer than 6 months.

#### Exclusion criteria

1. Age below 16 or above 25 years.
2. Active positive psychotic symptoms or history of first episode psychosis.
3. Severe learning disability problems (though mild to moderate learning difficulties will not be excluded).
4. Disease or physical problems likely to interfere with capacity to take part in interventions and assessments.
5. Non-English speaking to the degree that the participant is unable to fully understand and answer assessment questions or give informed consent.

### Recruitment and procedure

Three centres are involved in the study. Participants are drawn from secondary mental health care settings including outpatient youth mental health, early detection, and early intervention services, youth, social and educational services, and primary care mental health services in the three centres in East Anglia (Norfolk and Suffolk), Sussex and Greater Manchester. Potential participants from these services who consent to be contacted about the trial are referred by a professional to the trial team and provided with a trial information sheet. Research assistants (RAs) then meet with the potential participant in person to obtain written informed consent and perform the screening assessments (Fig. 1). Potential participants categorised as ‘screen failures’ are permitted to re-screen at a later date if appropriate. Potential participants eligible at screen are invited to complete the baseline assessment and, upon doing so, are randomised. All randomised participants are invited to undergo three post-randomisation follow-up assessments at 9, 15 and 24 months (Fig. 1). Interventions are provided from randomisation allocation until commencement of the 9 month post-randomisation follow-up assessments (Fig. 1).

### Sample size

The target sample size is 270 participants, providing 135 participants in each trial arm. The primary outcome is hours per week in structured activity on the time use survey [10, 54] at 15 months, which is likely not to follow a normal distribution but could have a positive skew. Analyses will therefore most likely use logarithmically transformed data. The sample size is based upon an effect size of 0.4 standard deviations being considered a minimum clinically significant benefit. A total of 270 participants would provide greater than 90% statistical power to detect a 0.4 standard deviation effect size; a total of 200 participants (i.e. even accounting for greater than 25% loss to follow-up) would provide 80% statistical power for the same effect size. A total of 100 participants were recruited in the internal pilot phase, with a target sample size of 170 to be recruited in the current non-pilot extension phase.

### Randomisation

Following pre-trial assessments, consenting participants are randomised to trial arms stratified by age in years (16–19, 20–25); site (Sussex, East Anglia, Manchester); severity of social disability (low functioning = 16 to 30 hours of structured activity per week, very low functioning = 0–15 hours of structured activity per week), and meeting symptomatic criteria for ARMS or not. A remote randomisation service assigns allocation to groups coordinated by the Norwich CTU. Allocation is by pre-set lists of permuted blocks with randomly distributed block sizes (agreed with the Trial Statistician). The lists are generated by the Data Management Team in Norwich CTU.

The allocation process is web-based, managed as part of the Trial Data Management System (TDMS). The sequence is hidden from TDMS users. Once allocated, the details are emailed to nominated individuals at the trial site to enable the allocation of treatment to be implemented. The allocation is not exposed to any other users of the database or other individuals.

Following completion and scoring of all baseline assessments per individual participant, site staff enter stratification information into the electronic database. Once submitted, an email is generated to the site staff to issue a participant number and inform that a participant has been randomised. Nominated members of the team (Trial Manager, Co-ordinators and Trial Therapists in the individual sites) receive an email detailing the allocation of said participant. This is logged by the Site Coordinator (locally) and Trial Manager (centrally) and the allocated Trial Therapists are informed to contact the participant (for those randomised to SRT plus ESC). Participants randomised to ESC alone are contacted by letter. The participant’s General Practitioner, referrer and usual care provider (if not the referrer) are informed of the allocation by a trial therapist or site co-ordinator. RAs do not have access to the allocation at any time during the trial.

### Blinding

Research staff collecting follow-up data are blinded to group allocation. This is maintained using a range of procedures. Following allocation, all participants in the trial, their relatives, their referrer and clinical team (if applicable) are asked not to reveal the allocation to the RAs. Participants, and any attending relatives or professionals if applicable, are also asked at the beginning of each assessment interview not to disclose the allocation. Outside of the assessments, RAs are shielded from discussion of participants in trial forums where the possibility of determining the allocation could occur. A system of web-based data entry ensures that RAs do not have access to information in the database that would reveal the allocation. Data entered into TDMS by trial therapists that might inadvertently lead to unblinding is hidden from non-trial therapist users. To test the success of blinding, the blind assessor is asked to guess the allocation group for each participant at the end of the final assessment.

All issues with blinding are recorded and reported to the site co-ordinator and trial manager. Reported issues with blinding are managed to maintain blind outcome assessments by reallocating ‘blind’ RAs to collect and score trial data. Promotion of ‘blind’ awareness and education continues throughout the trial, including communicating to administrative staff, referrers and clinical teams to minimise the occurrence of issues with the blind in addition to reminding participants and their relatives.

### Measurement quality

All assessors receive expert training in administration of outcome measures at least every 6 months. All assessors receive local site supervision in administration of outcome measurements for all participants screened at least weekly and central supervision with the trial manager at least fortnightly. Concordance for primary and key secondary outcome ratings is assessed every 2 months across all trial assessors, site co-ordinators and the trial manager in all three trial sites.

### Intervention adherence

All trial therapists receive expert training in intervention delivery at least every 6 months. All therapists receive local site supervision with site co-ordinators and separate central supervision at least monthly. Concordance ratings of intervention adherence and competence (using the Cognitive Therapy Rating Scale Revised [71]) are obtained from all trial therapists at least every 2 months.

### Statistical methods

The primary analysis will compare SRT plus ESC with ESC alone on hours spent in structured activity per week at 15 months post-randomisation. The primary analysis will be on the intention-to-treat principle, i.e. all participants will be followed up for data collection irrespective of adherence to treatment and will be analysed according to group allocation rather than intervention received. Assuming a normal (or corrected to normal) distribution, a linear model will be constructed. This will include recruiting site (as a random factor), weekly structured activity hours at baseline (as a covariate) and any factors considered prognostic and determined in advance of any analysis, together with treatment arm as a fixed effect.

The primary intention-to-treat analysis is intended to provide inferences regarding the effectiveness of the intervention overall, not to provide inferences regarding the causal effect of the intervention itself, but on the intervention as deployed in ‘real life’.

Statistical significance will be set at the conventional (two-tailed) 5% level and all parameter estimates will be presented with 95% confidence intervals. Analyses will be carried out by the trial statistician blinded to group identity (i.e. ‘subgroup’ blind). There are no plans for interim efficacy or subgroup analyses. Appropriate techniques to manage missing data will be used depending upon the nature and extent of missing data and analysis will also take account of clustering effects with regard to individual therapists. These and other more detailed aspects of the analysis plan are set out formally in the Statistical Analysis Plan held by the Norwich CTU.

The main measure of outcome in the economic analysis will be the EuroQol-5D-3 L [70]. This will enable a cost-utility analysis to be conducted, where the incremental Quality Adjusted Life Year gain associated with SRT compared to ESC will be estimated over the 15-month trial period. Costs will be estimated from the perspective of the NHS and PSS, including the cost of the intervention (based on information from service providers) and other NHS and PSS costs using a (self-reported) modified version of the client services receipt inventory [69]. Cost-effectiveness analyses will also be performed, where the effectiveness of SRT compared to ESC will also be assessed in relation to activity (time use) and symptoms (CAARMS). Analyses will be undertaken in order to estimate both the incremental cost and incremental effect associated with SRT compared to treatment as usual (this within-trial analysis will be undertaken for a 15-month follow-up period as part of the primary analysis, a 24-month follow-up analysis is also planned).

### Recruitment

#### Internal pilot phase

Between January 2013 and February 2014, 100 participants were recruited and randomised. By end of March 2016, 24 month follow-up assessment data had been collected from these participants. These 100 participants will be combined with those recruited in the substantive extension phase, providing the full sample.

#### Substantive non-pilot extension phase

The substantive extension target for recruitment is 170 participants recruited and randomised. Recruitment began September 2015.

## Discussion

This definitive trial will be the first to specifically address both social disability and mental health problems amongst a vulnerable and high-risk population of young people presenting with social disability and severe and complex non-psychotic mental health problems. This is a group who are underrepresented in research, underserved by current mental health services and treatments, and are at risk of lifelong social disability and severe mental health problems without effective intervention. The trial will provide an essential evidence base for developing interventions for young people with severe and complex mental health problems who have social disability characterised by inactivity in social and economic domains and social withdrawal. The primary follow-up assessments at post-treatment and at 15 months will be completed at the end of 2018 and the final 24-month follow-up will be completed in 2019.

### Protocol changes

The amendments made to the trial protocol since commencement of the trial are shown in Table 1.

**Table 1:** Changes to protocol

Area	Change
Trial documentation	Provision of crisis cards and summary report of assessments to participants
Measurement of outcome	Changes to assessment battery; addition of Structured Clinical Interview for Diagnostic and Statistical Manual of Mental Disorders, Version Four, reduction in neurocognitive battery, reduction from full CAARMS to short version, addition of the Alcohol Use Disorders Identification Test and the Drug Use Disorders Identification Test
Time point of measurement	Addition of 24-month follow-up assessment point
Sponsorship	Sussex Partnership NHS Foundation Trust replaced Norfolk and Suffolk NHS Foundation Trust as trial sponsor

No deviations from the protocol have been recorded

#### Trial status

Trial recruitment is active and ongoing.

## Additional file

Additional file 1: SPIRIT Checklist. (DOC 121 kb)

## Abbreviations

ARMS: at risk mental states
CAARMS: Comprehensive Assessment of At Risk Mental States
CBT: cognitive behavioural therapy
CTU: Clinical Trials Unit
ESC: enhanced standard care
NEET: not in employment, education and training
NHS: National Health Service
RA: research assistant
RCT: Randomised Controlled Trial
SRT: social recovery therapy
TDMS: trial data management system
UHR: ultra-high risk
